# Phytochemical Composition, Antibacterial, and Antibiotic-Resistance Modulatory Activity of Extracts of *Lippia multiflora* Moldenke*, Terminalia mollis* M. A. Lawson, and *Cinchona officinalis* L. Against Multidrug-Resistant *Pseudomonas aeruginosa*

**DOI:** 10.1155/sci5/3403280

**Published:** 2024-12-24

**Authors:** Richard Mouozong, Aimé Gabriel Fankam, Varelle Lambou Diffo, Valaire Yemene Matieta, Fabrice Junior Megaptche, Victor Kuete

**Affiliations:** Department of Biochemistry, University of Dschang, Dschang, Cameroon

**Keywords:** antibacterial, antibiotic, *Cinchona officinalis*, *Lippia multiflora*, multidrug-resistant, *Pseudomonas aeruginos*a, synergy, *Terminalia mollis*

## Abstract

*Pseudomonas aeruginosa* is a critical-class pathogen that shows great resistance to most conventional antibiotics. Hence, it is of utmost importance to search for novel drugs to fight infections caused by this bacterium. This study aimed to evaluate the antibacterial activity of *Lippia multiflora, Terminalia mollis,* and *Cinchona officinalis* extracts alone and in combination with antibiotics against multidrug-resistant (MDR) *P. aeruginosa*. Phytochemical analysis was performed using standard qualitative and quantitative assays. The microdilution method was used to assess the antibacterial and antibiotic-resistance modulatory activity of the extracts. The interaction between antibiotics and *Cinchona officinalis* leaf extract was carried out using the checkerboard broth microdilution method. Phenols and flavonoids were detected in all extracts, whereas other phytochemical classes were selectively distributed. *T. mollis* leaf extract demonstrated the highest phenolic content (151.59 mg GAE/g), while *L. multiflora* leaf (LML) extract showed the highest flavonoid content (24.51 mg QE/g). These extracts exhibited antibacterial activity, with minimum inhibitory concentrations (MICs) ranging from 128 to 2048 μg/mL. LML extract displayed the best antipseudomonal activity, with MIC of 128 μg/mL against ATCC 27853 and 256 μg/mL against some MDR isolates (PA1, PA2, and PA7). Moreover, *C. officinalis* leaf extract (MIC/8), although weakly active, had improved by 2 to 64-fold the activity of imipenem, streptomycin, kanamycin, and ceftriaxone against MDR *P. aeruginosa*. It also showed synergy (ΣFIC ≤ 0.5) with streptomycin, ampicillin, tetracycline, and vancomycin against *P. aeruginosa* PA3. The overall results indicate that the tested extracts, especially those from *L. multiflora* and *C. officinalis* leaves, necessitate further exploration for the development of natural drugs to treat infections caused by MDR *P. aeruginosa*.

## 1. Introduction

Infections are undoubtedly the major cause of death worldwide. Approximately 700,000 deaths occur annually due to microbial infections [1]. For more than three decades, microbial infections have shown recurrence, largely due to the gradual emergence of antibiotic-resistant pathogenic bacteria. This is primarily associated with the incorrect and overuse of antibiotics [2]. If no new antimicrobial strategies are discovered by 2050, antibiotic-resistant infections could lead to circa 10 million annual deaths on a global scale [3]. The ESKAPE pathogens, *Enterococcus faecium*, *Staphylococcus aureus*, *Klebsiella pneumoniae*, *Acinetobacter baumannii*, *Pseudomonas aeruginosa*, and *Enterobacter* spp., represent the greatest threats to human health because of their ability to evade antibiotic treatments through various mechanisms of resistance [4].

*P. aeruginosa* is a bacterial strain at blame for most of nosocomial infections. It is implicated in bacteremia, respiratory tract infections, urinary tract infections, and wound infections [5–7]. This species is classified as “critical” on the World Health Organization's (WHO) priority list of bacterial pathogens for which research and development of new antibiotics are in dire need [8, 9]. *Pseudomonas* species, especially the opportunistic pathogen *P. aeruginosa*, are documented to be multidrug-resistant (MDR) to antibiotics, specifically aminoglycosides, fluoroquinolones, and *β*-lactams. They exhibit remarkable resistance mechanisms such as overexpression of efflux pumps, production of antibiotic-inactivating enzymes, reduced expression of porins, acquisition of resistance genes, and mutation of quinolone targets [10]. As a result, in addition to monitoring antibiotic resistance, substituting the arsenal of anti-infective molecules has become a priority.

Medicinal plants have been used by approximately 80% of the population to treat various health problems, including infections [11]. Even today, there are large areas of the world that do not have access to this modern medicine that still use traditional medicine that involves the direct use of medicinal plants [12]. Therefore, medicinal plants could offer an alternative to antibiotic resistance. Several studies have demonstrated that plant extracts have a remarkable ability to combat bacteria, particularly MDR bacteria, either alone or in combination with antibiotics [13–19]. Previous studies have reported the medicinal uses, the phytochemistry as well as the evidence of the biological activity of *Lippia multiflora* Moldenke (Verbenaceae), *Terminalia mollis* M. A. Lawson (Combretaceae), and *Cinchona officinalis* L. (Rubiaceae) (Table 1). These studies were mainly based on the antibacterial activity of extracts from the roots or bark of these plants obtained with different solvents on others bacteria species than *Pseudomonas* and were limited to the determination of MIC or the diameter of the zone of inhibition. Moreover, it is the first time to evaluate the interaction effects of the tested extracts with antibiotics against drug resistant bacteria.

This study was designed to evaluate the antibacterial potential of the leaf extracts of these plants alone and in combination with antibiotics against MDR *P. aeruginosa*. Moreover, their phytochemical composition was also evaluated in this study.

## 2. Materials and Methods

### 2.1. Plant Materials and Extraction

The plant materials investigated in this study were leaves from three Cameroonian medicinal plants, namely, *L. multiflora, T. mollis,* and *C. officinalis*, collected in August 2022 at Mbouda and Dschang, West Region of Cameroon. These plants were identified under specific reference numbers at the National Herbarium of Cameroon in Yaoundé (Table 1). The collected plants were cleaned, dried in the absence of UV radiation, and ground. The obtained powders (0.1 kg) were macerated in methanol 95% in a 1/3 (w/v) ratio for 48 h, at room temperature (with constant agitation). Then, the mixtures were filtered using Whatman filter paper No. 1, and the filtrates were concentrated at 65°C using a rotary evaporator (BÜCHI R-200). The crude extracts were collected and dried in an oven at 40°C for complete solvent evaporation and stored at 4°C for further use.

### 2.2. Chemicals for Antibacterial Assays

Nine commonly used antibiotics, including doxycycline (DOX), kanamycin (KAN), ceftriaxone (CEF), ciprofloxacin (CIP), tetracycline (TET), streptomycin (STR), vancomycin (VAN), imipenem (IMI), and ampicillin (AMP) (Sigma-Aldrich), were used. p-Iodonitrotetrazolium chloride (INT) 0.2% (Sigma-Aldrich) was used as the bacterial growth indicator, and dimethyl sulfoxide (DMSO) was used to dissolve the plant extracts.

### 2.3. Microbial Strains and Culture Media

Ten *P. aeruginosa* including one reference strain from the American Type Culture Collection (ATCC 27853) and nine MDR clinical isolates (PA1, PA2, PA3, PA4, PA7, PA9, PA12, PA14, and PA124) were used in this study. Their resistance features were previously reported [29, 30]. Mueller–Hinton agar and Mueller–Hinton broth (MHB) (Accumix, India) were used for bacterial culture and antibacterial assays, respectively.

### 2.4. Determination of the Phytochemical Composition of Extracts

#### 2.4.1. Phytochemical Screening of Extracts

The plant extracts underwent phytochemical screening in order to detect the presence of important secondary metabolite classes, including alkaloids, anthocyanins, flavonoids, phenols, saponins, tannins, and triterpenes. This screening was performed using established phytochemical methods as described by Tiwari et al. [31]. The assays were visually observed for change in color or formation of precipitate after the addition of specific reagents.

#### 2.4.2. Determination of the Total Phenolic Content (TPC) of Extracts

The TPC of the various extracts was determined using the spectrophotometric method with the Folin–Ciocalteu reagent [32]. The reaction mixture consisted of 0.02 mL of extract (2 mg/mL), 0.2 mL of 2 N Folin–Ciocalteu reagent, and 0.4 mL of 20% sodium carbonate solution. The mixture was agitated and incubated at 40°C in a water bath for 20 min, and then, the absorbance was measured at 760 nm and expressed in milligram of gallic acid equivalent per gram of dry extract (mg GAE/g) using the gallic acid standard curve. Each sample was assayed in triplicate.

#### 2.4.3. Determination of Total Flavonoid Content (TFC) of Extracts

The quantification of TFC of the tested extracts was carried out using the aluminum trichloride method [33]. In summary, 100 μL of the extract (2 mg/mL) was mixed with 1.49 mL of distilled water and 30 μL of 5% NaNO_2_. After 5 min of incubation at room temperature, 30 μL of 10% AlCl_3_ was added. Following an additional 6 min incubation, 200 μL of 0.1 M NaOH and 240 μL of distilled water were introduced. After thorough mixing, the absorbance was measured at 510 nm and converted to milligram of quercetin equivalents per gram of dry extract (mg QE/g) using the quercetin standard curve. Each sample was analyzed in triplicate.

### 2.5. Evaluation of the Antibacterial Activity of Extracts

The minimum inhibitory concentration (MIC) and minimum bactericidal concentration (MBC) of the plant extracts were determined by the microdilution method using INT calorimetric assays [29, 34]. Briefly, the tested samples were dissolved in DMSO/MHB and added in the first lines of a 96-well microplate containing MHB, then serially diluted twice. This was followed by the addition of 100 μL of inoculum (10^6^ CFU/mL) to each well. The final concentrations of extracts and antibiotics ranged from 2048 to 16 μg/mL and from 256 to 2 μg/mL, respectively. Imipenem was used as the reference antibiotic. The MICs of the samples were detected after 18 h of incubation at 37°C, following the addition of 40 μL of INT (0.2 mg/mL) and incubation at 37°C for 30 min. The MIC of each sample was defined as the lowest concentration of the sample that completely prevented bacterial growth (absence of pink coloration in the wells). The MBC of the sample was determined by subculturing 50 μL of the suspensions from the wells that did not exhibit any growth after the MIC assay into 150 μL of fresh MHB, followed by a reincubation at 37°C for 48 h. The MBC was defined as the lowest concentration of the samples that did not produce coloration after the addition of INT [29]. Each sample (extract or antibiotic) was tested in duplicate, and the experiment was repeated thrice.

### 2.6. Modulation Assay

To assess the antibiotic modulating effects of plant extracts, the MICs of antibiotics were determined in the presence and absence of plant extracts using the liquid microdilution method as previously described [35, 36]. Briefly, antibiotics were serially diluted as described in Section 2.5., and the plant extract was added at its subinhibitory concentration (MIC/8). Subsequently, inoculum was introduced into the wells, and the MICs were determined as above. Each experiment was done duplicate and repeated thrice. The modulation factor (MF) was calculated as the ratio of the MIC of the antibiotic alone to that of the antibiotic in the presence of the extract: MF = (MIC antibiotic)/(MIC antibiotic + extract). A MF ≥ 2 was set as the threshold for the biological significance of antibiotic potentiation effects [37].

### 2.7. Evaluation of the Effects of Interaction Between Antibiotics and *C. officinalis* Leaf Extract

The interactions between antibiotics and extract of *C. officinalis* were investigated using the checkerboard broth microdilution method [38]. For this, antibiotic was added and diluted along the *x*-axis, while the extract was added and diluted along the *y*-axis. After this, the final volume of each well was 100 μL. Subsequently, 100 μL of inoculum containing 2 × 10^6^ CFU/mL of bacteria was added to wells. The plates were sealed and incubated for 18 h at 37°C and MIC detected as described above. All experiments were done in triplicate. The fractional inhibitory concentration index (∑FIC) was calculated as ∑FIC = FIC of extract + FIC of antibiotic, where FIC of extract = MIC of extract in combination/MIC of extract alone and FIC of antibiotic = MIC of antibiotic in combination/MIC of antibiotic alone. The results were interpreted as follows: synergy (ƩFIC ≤ 0.5); additivity (0.5 < ƩFIC ≤ 1); indifferent if (ƩFIC > 1–2); and antagonism (ƩFIC > 2) [39].

### 2.8. Statistical Analysis

Data for TPC and TFC were reported as mean with the standard deviation (mean ± SD). Statistical analysis was performed using GraphPad Prism for windows, version 5.0.1. One-way analysis of variance (ANOVA) followed by Tukey's multiple comparison test was performed to compare the means. Results were considered significant when *p* < 0.05.

## 3. Results

### 3.1. Phytochemical Composition of Extracts

#### 3.1.1. Qualitative Phytochemical Composition

The results of the phytochemical studies indicated that all the tested extracts contained flavonoids and phenols. Alkaloids, triterpenes, saponins, anthocyanins, and tannins were selectively present in extracts. Moreover, all the studied phytochemicals were detected in *T. mollis* leaf extract (Table 2).

#### 3.1.2. TPC of Extracts

**Figure 1:**
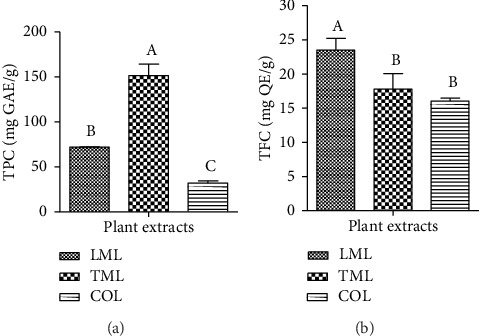
Chemical composition of the plant extracts. (a) Total phenolic content; (b) total flavonoid content. LML: *Lippia multiflora* leaf; TML: *Terminalia mollis* leaf; COL: *Cinchona officinalis* leaf; TPC: total phenolic content; TFC: total flavonoid content; GAE: gallic acid equivalent; QE: quercetin equivalent. Values followed by different letters are significantly different (one-way ANOVA with Tukey's test, *n* = 3, *p* < 0.05).

The results presented in Figure 1(a) show that the extract of *T. mollis* had the highest TPC (151.59 mg GAE/g), which was significantly (*p* < 0.05) higher than that of the other extracts. *C. officinalis* leaf extract exhibited the lowest TPC.

#### 3.1.3. TFC of Extracts

The results revealed a TFC of *L. multiflora* leaf (LML) extract significantly (*p* < 0.05) higher than that of all the other extracts (24.51 mg QE/g). Moreover, *C. officinalis* leaf extract presented the lowest TFC compared to the other extracts (Figure 1(b)).

### 3.2. Antibacterial Activity

The results revealed varying antibacterial activities for the tested extracts, with MICs ranging from 128 to 2048 μg/mL (Table 3). Additionally, *T. mollis* and LML extracts were active on all the tested *P. aeruginosa* strains (100%), whereas *C. officinalis* leaf extract showed the lowest spectrum of activity (40%). Furthermore, LML extract demonstrated tremendous antipseudomonal activity (MIC = 128 μg/mL) against ATCC 27853 and very good activity (MIC = 256 μg/mL) against clinical isolates, PA 1, PA 2, and PA 7. The reference antibiotic, imipenem, was active against all the tested *P. aeruginosa* strains, with MICs ranging from 4 to 256 μg/mL. As for the MBCs, they were generally greater than 2048 μg/mL for the extracts and 256 μg/mL for imipenem.

### 3.3. Resistance Modulatory Activity

A preliminary test at subinhibitory concentration (MIC/8) was conducted against the MDR *P. aeruginosa* (PA124), considered as one of the most resistant MDR bacterium allowed the selection of *C. officinalis* leaf extract for the evaluation of its antibiotic-resistance modulatory activity against five other MDR *P. aeruginosa* isolates. The results obtained indicate 2- to 64-fold improvement of the activity of antibiotics in the presence of *C. officinalis* leaf extract (Table 4). This extract has potentiated the activity of all the tested antibiotics, especially that of STR on 5/6 (83.33%); KAN on 4/6 (66.67%); imipenem and TET on 3/6 (50%); CIP, DOX, CEF, AMP, and VAN on 2/6 (33.33%) of the tested bacteria.

### 3.4. Interaction Effects Between Antibiotics and Extract of *C. officinalis*

Table 5 presents the results of the interactions between *C. officinalis* leaf extract and some commonly used antibiotics (STR, TET, AMP, and VAN) against *Pseudomonas aeruginosa* PA3. They show that all the combinations of *C. officinalis* with the antibiotics exhibited synergy (∑FIC ≤ 0.5) against the PA3 clinical isolate. A strong synergy was observed with STR (∑FIC = 0.25).

## 4. Discussion

Regarding the expansion of MDR bacteria, there is an urgent need of novel substances in antibiotic therapy to tackle global infectious diseases. Medicinal plants represent a potential source of antimicrobial agents used in the treatment of infectious diseases [40, 41]. According to Tankeo and Kuete [42], the tested extracts exhibited weak (MIC > 1024 μg/mL) to excellent (32 < MIC ≤ 128 μg/mL) antipseudomonal activities. This variability in activity could be attributed to the differences in the observed phytochemical compositions between the extracts [41]. LML extract demonstrated excellent antipseudomonal activity (MIC = 128 μg/mL) against ATCC27853 and very good activity (128 < MIC ≤ 256 μg/mL) against PA1, PA2, and PA7. This result aligns with those of Samba et al., who reported antibacterial activity of *L. multiflora* essential oil against *Staphylococcus aureus*, *Escherichia. coli*, and *P. aeruginosa*. The authors ascribed this activity to the presence of limonene, a compound that belongs to the class of monoterpenes [21]. It has been reported that hydrophobic constituents, such as terpenes detected in the most active extract, exhibit antibacterial activity by disrupting the bacterial membrane through lipid peroxidation, followed by the leakage of intracellular materials, causing cell death [43, 44]. Interestingly, this study highlights for the first time the antibacterial activity of *L. multiflora* extract against resistant *P. aeruginosa*. Júnior et al. [25] demonstrated that *T. mollis* extract had moderate activity against six bacterial strains, including *P. aeruginosa*. The same observation was noted in this study. These activities can be explained by the chemical composition of these plant extracts, which contain high phenolic content, as detected in our work [40, 45]. The antimicrobial activity of phenols may be attributed to the iron deprivation or hydrogen bonding with vital proteins, such as microbial enzymes [40, 46]. Despite a lower number of phytochemical classes detected in LML extract (4 out of 7), it displayed better activity. These observations clearly confirm that the activity of a plant extract depends not only on the presence of bioactive compounds but also on their quantities and potential interactions with other constituents present in the extract [47].

A promising approach to overcome MDR bacteria involves combining plant extracts with antibiotics in other to discover resistance modulators such as efflux pump inhibitors or compounds having synergistic effect with antibiotic [48–51]. In this study, we evaluated the synergistic effects of three plant extracts with nine antibiotics to enhance their activity against antibiotic-resistant isolates of *P. aeruginosa*. Generally, we observed an increment in the activity of antibiotics when combined with our extracts, particularly with the less active one, *C. officinalis* leaf extract. It demonstrated significant modulatory activity (MF between 2- and 64-fold) with AMP, TET, STR, KAN, and VAN, mainly against *P. aeruginosa* PA3 and PA12. These observations suggest that the components present in the *C. officinalis* leaf extract may act by inhibiting the expression of efflux pumps, which is the primary mechanism of resistance in *P. aeruginosa* [52]. According to the European Committee for Antimicrobial Susceptibility Testing (EUCAST) criteria [39], *C. officinalis* leaf extract presents a synergistic interaction (∑FIC ≤ 0.5) with TET, STR, AMP, and VAN against PA3. This suggests that secondary metabolites detected in *C. officinalis* leaf extract such as triterpenes or phenols may act on different sites than the above antibiotics, contributing to the rapid bacterial lysis.

## 5. Conclusions

Our results provide essential information on the potential use of the studied plant extracts, mainly LML extract for the treatment of infections caused by MDR *P. aeruginosa*. Beyond their antibacterial properties, the data suggest that combining the extract of *C. officinalis* with various antibiotics could be a valuable approach to managing bacterial infections associated with MDR *P. aeruginosa*. The study was based on the 95% methanol extracts. Thus, further investigations using different solvents of various polarities will be required to identify lead compounds for the development of appropriate antipseudomonal drugs. Moreover, in vivo antibacterial and toxicity study of the most active extracts will be necessary to encourage their further use.

## Figures and Tables

**Table 1:** Informations on the studied plants and the evidence of their biological activities.

Plant (family)	Common/local name	Part used	Identification number	Traditional uses	Isolated compounds	Bioactivities
Lippia multiflora moldenke (verbenaceae)	Gambian tea bush/ligi or gossolderi or fever tea	Leaf	77532/SRF/Cam	Liver failure, jaundice, stomach ache, lung infections, fever, oral candidiasis [20]	Limonene, piperitenone, neral, citral, elemol, p-cymene, transtagetone, and artemisia ketone [21]	Antimicrobial activity of essential oil: Sa, Ef, and Pa [21]
Terminalia mollis M. A. Lawson (combretaceae)	Opok, black afara	Leaf	64212/HNC	Diarrhea, gonorrhea, malaria, and HIV treatment supplement [22]	Arjunolic acid, 2*α*, 3*β*, 23-trihydroxyurs-12-en-28-oic acid, 2*α*-hydroxyursolic acid, gallic acid, and chebulanin [23]	Antimicrobial activity of methanol and aqueous extracts of roots: Sa, Ec, Pa, Kp, St, Ba, Ca, Cn, and Af [22]; antitrypanosomal activity of root methanol and aqueous extracts: Growth inhibition of *Trypanosoma brucei* brucei with an IC_50_ value of 3.72 μg/mL [24]
Cinchona officinalis L. (rubiaceae)	Quinine plant, fever tree	Leaf	12431/HNC/SRF	Diarrhea, dysentery, fever, malaria [25]	Resorcylic acid, cinnamate, limonene, camphor, quinine, dihydroquinine, cinchonidine, epiquinine, quinidine, dihydroquinidine, cinchonine [27]	Antimicrobial activity of aqueous extract of barks: Sa ATCC 6538 [27]; cytotoxicity of bark methanol extract: significantly active with an IC_50_ value of 9 μg/mL at 48 h, on breast cancer cells MCF-7 [28]

Abbreviations: Af, *Aspergillus flavus*; Ba, *Bacillus anthracis*; Bs, *Bacillus subtilis*; Ca, *Candida albicans*; Cam, Cameroon; Cn, *Cryptococcus neoformans*, Ec, *Escherichia coli*; Ef, *Enterococcus faecalis*; HNC, Cameroon National Herbarium; Kp, *Klebsiella pneumonia*; Pa, *Pseudomonas aeruginosa*; Sa, *Staphylococcus aureus*; SRF: Société des reserves forestières; St, *Salmonella typhimurium*.

**Table 2:** Extraction yields, aspects, and phytochemical composition of the plant extracts.

Plant extracts	Part used	Phytochemical composition						
		Alkaloids	Triterpenes	Saponins	Phenols	Flavonoids	Tannins	Anthocyanins
Lippia multiflora	Leaf	+	+	−	+	+	−	−
Terminalia mollis	Leaf	+	+	+	+	+	+	+
Cinchona officinalis	Leaf	+	+	+	+	+	−	−

*Note:* (+): present; (−): absent.

**Table 3:** MIC and MBC in μg/mL of the plant extracts and imipenem against *P. aeruginosa.*

Bacteria	*T. mollis* leaf		*L. multiflora* leaf		*C. officinalis* leaf		Imipenem	
	MIC	MBC	MIC	MBC	MIC	MBC	MIC	MBC
ATCC27853	1024	—	128	—	512	—	16	256
PA124	2048	—	512	—	512	—	32	> 256
PA14	512	—	1024	—	2048	—	256	> 256
PA12	1024	—	512	1024	—	—	128	256
PA9	2048	—	512	—	—	—	4	> 256
PA7	2048	—	256	1024	—	—	4	> 256
PA4	2048	—	1024	—	—	—	16	> 256
PA3	2048	—	512	—	—	—	16	> 256
PA2	512	—	256	—	2048	—	16	> 256
PA1	2048	—	256	—	—	—	16	> 256

*Note:* —: MIC and MBC not detected up to 2048 μg/mL

Abbreviations: MBC, minimal bactericidal concentration; MIC, minimal inhibitory concentration.

**Table 4:** Antibiotic resistance modulatory activity of *C. officinalis* leaf extract against MDR *P. aeruginosa*.

Antibiotics	Extracts' concentration	Bacteria, MIC (μg/mL), and modulating factors (in bracket)						Modulating effect (%)
		ATCC27853	PA 14	PA12	PA3	PA1	PA124	
CIP	0 MIC/8	1 0.5 (**2**)	2	≤ 0.5	≤ 2	1	> 32	33.33
			1 (**2**)	≤ 0.5 (nd)	≤ 2 (nd)	1 (1)	> 32 (nd)	

IMI	0	8	8	≤ 2	≤ 2	16	32	**50**
	MIC/8	≤ 2 (**≥ 4**)	4 (2)	≤ 2 (nd)	8 (≤ 0.25)	8 (**2**)	64 (0.5)	

DOXI	0	64	64	32	≤ 2	64	128	33.33
	MIC/8	64 (1)	64 (1)	≤ 2 (**≥ 16**)	≤ 2 (nd)	64 (1)	32 (**4**)	

STR	0	8	32	≤ 2	8	32	256	**83.33**
	MIC/8	4 (**2**)	16 (**2**)	≤ 2 (nd)	≤ 2 (≥ **4**)	16 (**2**)	64 (**4**)	

KAN	0	> 256	256	256	256	256	16	**66.67**
	MIC/8	> 256 (nd)	32 (**8**)	4 (**64**)	32 (**8**)	64 (**4**)	32 (0.5)	

TET	0	32	256	32	16	256	128	**50**
	CMI/8	32 (1)	256 (1)	4 (**8**)	4 (**4**)	256 (1)	64 (**2**)	

CEF	0	64	32	≤ 2	8	16	> 256	33.33
	MIC/8	32 (**2**)	8 (4)	≤ 2 (nd)	16 (0.5)	16 (1)	256 (> 1)	

AMP	0	512	—	—	256	—	—	33.33
	MIC/8	512 (1)	—	8 (**≥ 64**)	16 (**16**)	—	—	

VAN	0	—	—	4	512	—	—	33.33
	MIC/8	—	—	≤ 2 (**≥ 2**)	8 (**64**)	512 (nd)	—	

*Note:* —: undetectable MIC up to 512 μg/mL; (): modulation factor; nd: not determined; values in bold represent modulation factors ≥ 2 and a modulatory effect observed in at least 50% of the tested bacteria.

Abbreviations: AMP, ampicillin; CEF, ceftriaxone; CIP, ciprofloxacin; DOX, doxycycline; IMI, imipenem; KAN, kanamycin; PA, *Pseudomonas aeruginosa*; STR, streptomycin; TET, tetracycline; VAN, vancomycin.

**Table 5:** Fractional inhibitory concentrations (FIC) of the combinations of antibiotics and *C. officinalis* leaf extract against *P. aeruginosa* PA3.

Antibiotic	FIC_extract_	FIC_antibiotic_	ƩFIC	Interaction
STR	0.125	0.125	0.25	Synergy
TET	0.125	0.25	0.375	Synergy
AMP	0.125	0.25	0.375	Synergy
VAN	0.25	0.25	0.5	Synergy

Abbreviations: AMP, ampicillin; FIC, fractional inhibitory concentrations; STR, streptomycin; TET, tetracycline; VAN, vancomycin.

## Data Availability

The data will be made available upon reasonable request through the corresponding author.
